# Effects of β-Nicotinamide Mononucleotide, Berberine, and Cordycepin on Lipid Droplet Content and Developmental Ability of Vitrified Bovine Oocytes

**DOI:** 10.3390/antiox12050991

**Published:** 2023-04-24

**Authors:** Xi Xu, Baigao Yang, Hang Zhang, Xiaoyi Feng, Haisheng Hao, Weihua Du, Huabin Zhu, Adnan Khan, Muhammad Zahoor Khan, Peipei Zhang, Xueming Zhao

**Affiliations:** 1 Institute of Animal Sciences (IAS), Chinese Academy of Agricultural Sciences (CAAS), No.2 Yuanmingyuan Western Road, Haidian District, Beijing 100193, China; 2 Key Laboratory of Animal Genetics, Breeding and Reproduction, MARA, National Engineering Laboratory for Animal Breeding, College of Animal Science and Technology, China Agricultural University, Beijing 100193, China

**Keywords:** oocytes, vitrification, bovine, NMN, BER, COR

## Abstract

Oocyte vitrification is crucial for livestock reproduction, germplasm conservation, and human-assisted reproduction, but the overabundance of lipids is highly detrimental to oocyte development. It is necessary to reduce the lipid droplet content of oocytes before cryopreservation. This study analyzed the impact of β-nicotinamide mononucleotide (NMN), berberine (BER), or cordycepin (COR) on various aspects of bovine oocytes, including lipid droplet content and the expression levels of genes related to lipid synthesis in bovine oocytes, development ability, reactive oxygen species (ROS), apoptosis, and the expression levels of genes associated with endoplasmic reticulum (ER) stress, and mitochondrial function in vitrified bovine oocytes. The results of our study indicated that 1 μM NMN, 2.5 μM BER, and 1 μM COR were effective in reducing the lipid droplet content and suppressing the expression levels of genes involved in lipid synthesis in bovine oocytes. Our findings showed that the vitrified bovine oocytes treated with 1 μM of NMN had a significantly higher survival rate and better development ability compared to the other vitrified groups. Additionally, 1 μM NMN, 2.5 μM BER, and 1 μM COR decreased the levels of ROS and apoptosis, decreased the mRNA expression levels of genes involved in ER stress and mitochondrial fission but increased the mRNA expression levels of genes associated with mitochondrial fusion in the vitrified bovine oocytes. Our study results suggested that 1 μM NMN, 2.5 μM BER, and 1 μM COR effectively decreased the lipid droplet content and enhanced the development ability of vitrified bovine oocytes by lowering ROS levels, reducing ER stress, regulating mitochondrial function, and inhibiting apoptosis. Furthermore, the results showed that 1 μM NMN was more effective than 2.5 μM BER and 1 μM COR.

## 1. Introduction

Oocyte vitrification has proven to be a highly advantageous method in breeding livestock and preserving oocyte banks [1]. It boosts breeding efficiency [2] and helps to protect the genetic diversity of endangered animal species at a lower cost [3]. Furthermore, this method reduces the environmental impact and enhances animal welfare by minimizing the need for live animal transportation [4]. Meanwhile, the vitrification of oocytes helps to maintain the fertility of women suffering from illnesses [5]. Consequently, there has been rapid advancement in the development of vitrification technology for animal oocytes, including those from bovine [6], sheep [6], porcine [6], and equine [7] animals.

However, the viability of vitrified oocytes is significantly decreased due to damages caused by cryopreservation [8], which restricts the use of cryopreservation techniques. Domestic animals’ oocytes are very rich in lipids [9], especially those produced through in vitro methods [10,11], which are known to negatively impact cryosurvivability [12,13]. This is because lipid phase transformation and lipid peroxidation during cooling can cause damage to the membrane, endoplasmic reticulum (ER), and result in mitochondrial dysfunction [13]. Hara [14] reported that the developmental ability of cryopreserved porcine oocytes could be improved by removing lipids after centrifugation. Despite the potential benefits, centrifugation and micromanipulation are considered too complex and time-consuming for commercial use [15] and can negatively impact the subsequent development of oocytes [16], limiting their widespread application. As a result, researchers are focusing on using substances to enhance lipid metabolism, decrease lipid droplet content, and improve the cryopreservation outcome.

Recently, chemicals such as β-nicotinamide mononucleotide (NMN), berberine (BER), and cordycepin (COR) have garnered interest due to their potent regulatory functions in metabolism and stress resistance. NMN is a bioactive nucleotide that is a precursor to the synthesis of nicotinamide adenine dinucleotide (NAD) in mammalian cells [17]. It has been shown to participate in crucial physiological processes, including lipid metabolism [18], ER stress response [19], antioxidative, anti-apoptotic [20], DNA damage repair, and gene expression [21,22]. BER, a member of the isoquinoline alkaloids group, has a crucial role in reducing lipids, antioxidant, anti-inflammatory, and glucose levels [23]. As a key bioactive component derived from Cordyceps militaris, COR is known for its ability to inhibit lipid accumulation, inflammation, apoptosis, and oxidative stress [24,25]. Numerous studies have demonstrated that NMN, BER, and COR could effectively decrease lipid accumulation in mammalian cells [26,27,28,29] and enhance the development of oocytes in pigs [30,31,32] or mice [33,34]. However, there is limited information on the effect of NMN, BER, and COR on the vitrification of bovine oocytes, and even less research on the underlying mechanisms.

Therefore, this current study was aimed to investigate the impact of NMN, BER, or COR on the lipid droplet content and developmental potential of vitrified bovine oocytes. Additionally, the levels of ROS and apoptosis and mRNA expression levels of genes associated with lipid metabolism (*sterol regulatory element-binding protein 1*, *SREBP1*; *fatty acid binding protein 3*, *FABP3*; *peroxisome proliferator activated receptor γ*, *PPARG*), ER stress (*X-box binding protein 1*, *XBP1*; *activating transcription factor 4*, *ATF4*; *glucose regulatory protein 78*, *GRP78*), mitochondrial function (*mitofusin 1*, *MFN1*; *mitofusin 2*, *MFN2*; *fission mitochondrial 1*, *FIS1*; *dynamin related protein 1*, *DRP1*), antioxidant (*catalase*, *CAT*; *glutathione peroxidase 1*, *GPX1*; *superoxide dismutase 1*, *SOD1*), and apoptosis (*BCL2 associated X*, *BAX*; *BCL2 apoptosis regulator*, *BCL2*) were evaluated to understand the underlying mechanism of action of NMN, BER, or COR.

## 2. Materials and Methods

All chemicals and reagents used, unless specified otherwise, were obtained from Sigma-Aldrich Chemical Company (St. Louis, MO, USA). The plastic products were sourced from Thermo Fisher Scientific Company (Waltham, MA, USA). All animal processing conformed to guidelines developed by the Institutional Animal Care and Use Committee of the Chinese Academy of Agricultural Sciences.

### 2.1. Oocyte Collection and In Vitro Maturation (IVM)

Bovine ovaries were transported from the slaughterhouse to the laboratory within 2 h at 35 °C. Follicles of 2–8 mm were selected to obtain cumulus–oocyte complexes (COCs), and COCs wrapped in at least three intact layers of cumulus cells (CCs) were cultured separately in the IVM solution supplemented with NMN, BER, or COR. For IVM, at least 50 COCs were put into 500 μL IVM solution covered by oil at 38.5 °C, 5% CO_2_, for 22–24 h. The IVM solution was comprised of M199 (Gibco BRL, Grand Island, NY, USA), luteinizing hormone (10 μg/mL), estradiol (1 μg/mL), follicle-stimulating hormone (10 μg/mL), heparin (10 μg/mL), penicillin and streptomycin (0.4 mg/mL) (Gibco BRL, Grand Island, NY, USA), and fetal bovine serum (FBS, 10% *v*/*v*). After IVM, COCs were incubated with hyaluronidase (0.1%, *w*/*v*) to isolate CCs, and the oocytes exhibiting even distribution of cytoplasm and at the MII stage were selected for subsequent experiments.

### 2.2. Vitrification and Warming

According to the method described by Zhao [35] with slight modifications, the open-pulled straw (OPS) method was used to vitrify oocytes. For vitrification and warming, the pretreatment solution consisted of Dulbecco’s phosphate-buffered saline (DPBS) supplemented with ethylene glycol (EG,10%, *v*/*v*), dimethyl sulfoxide (DMSO, 10%, *v*/*v*), and FBS (10%). The vitrification solution was comprised of EG (20%, *v*/*v*) and DMSO (20%, *v*/*v*) in DPBS medium with FBS (20%, *v*/*v*), Ficoll (300 g/L), and sucrose (0.5 M). For the procedure of vitrification, oocytes were treated with the pretreatment solution for 30 s, delivered to the vitrification medium, aspirated into an OPS tube, and put in liquid nitrogen (LN_2_) within 25 s. As for warming, the vitrified oocytes were extracted from the LN_2_, immediately incubated in 0.5 M sucrose for 5 min and then in 0.25 M sucrose for another 5 min.

### 2.3. IVF Procedure of Oocytes

After 22–24 h IVM, fertilization was performed according to Brackett and Oliphant [36]. The fertilization medium was prepared by Brackett and Oliphant (BO) medium, bovine serum albumin (BSA) (20 mg/mL), heparin (20 μg/mL), penicillin (100 IU/mL), and 100 μg/mL streptomycin (100 μg/mL). Briefly, the frozen sperm were thawed at 38 °C, washed twice in BO medium, and resuspended and diluted to a concentration of 1 × 10^6^/mL with fertilization medium. For fertilization, 20–30 oocytes were transferred to 100 μL fertilization medium covered with mineral oil for 16–18 h, at 38.5 °C and 5%CO_2_. The hypothetical zygotes were stripped of CCs and cultured in Charles Rosenkrans medium with amino acids (CR1aa)solution for 48 h. The cleaved embryos were transferred to the CR1aa medium with 10% FBS for another 120 h to blastocysts. Half of the solution was replaced every 48 h during the culture period, and the cleavage rate and blastocyst rate were counted on day 2 and 7 after fertilization.

### 2.4. qRT-PCR Procedure

At least 50 oocytes were collected from each pool for RNA extraction by TriZol reagent (Invitrogen, Carlsbad, CA, USA). RNA was kept at −80 °C until use. According to the product manuals, the qRT-PCR procedure was carried out by an ABI 7500 SDS instrument (Applied Biosystems, Waltham, MA, USA) with the comparative Ct (2^−ΔΔ^Ct) method [37]. The primers used in the present study are shown in Table 1, and *β-ACTIN* was utilized as the reference gene.

### 2.5. ROS Staining of Oocytes

The ROS staining of bovine oocytes was carried out according to the method described by Zhao [35]. Briefly, oocytes were stained with 10 mM DCHFDA (Beyotime, Shanghai, China) for 20 min, then washed twice, and observed on the epifluorescence inverted microscope (Nikon, Tokyo, Japan) connected to a DSRi1 camera (Nikon, Tokyo, Japan). EZ-C1 Free Viewer software (Nikon, Tokyo, Japan) was utilized for fluorescence intensity.

### 2.6. Lipid Droplets Staining of Oocytes

With slight modifications according to the previous method [38], oocytes were washed in 0.1% PVA-PBS, incubated with 10 μg/mL Nile red solution (Solarbio, Beijing, China) for 10 min, and observed under the epifluorescence inverted microscope (Nikon, Tokyo, Japan) connected to a DSRi1 camera (Nikon, Tokyo, Japan). Fluorescent images were analyzed by the EZ-C1 Free Viewer software (Nikon, Tokyo, Japan).

### 2.7. PS Externalization of Oocytes

An Annexin V-FITC apoptosis detection Kit (Beyotime, Shanghai, China) was applied to measure the externalization of PS in bovine MII oocytes [35]. Oocytes were incubated in 200 μL binding buffer consisting of 10 μL of propidium iodide (PI) and 5 μL of Annexin V-FITC for 5–10 min and then examined under the epifluorescence inverted microscope (Nikon, Tokyo, Japan) connected to a DSRi1 camera (Nikon, Tokyo, Japan). According to the method described by Anguita [39], oocytes were classified into three groups based on staining signals of Annexin V, including (i) early apoptotic oocytes with an obvious Annexin V-positive signal in the membrane, (ii) survival oocytes with no Annexin V staining, and (iii) necrotic oocytes showing PI-positive red nuclei.

### 2.8. Experimental Design

Firstly, the IVM medium was supplemented with different concentrations of NMN (0.1 μM, 1 μM, 10 μM, 100 μM), BER (1.25 μM, 2.5 μM, 5 μM, 10 μM), or COR (0.1 μM, 1 μM, 10 μM, 100 μM) to detect the effect of the maturation of bovine oocytes and the reduction of lipid droplet content on the survival rate and development ability of bovine vitrified oocytes. Subsequently, the effects of 1 μM NMN, 2.5 μM BER, and 1 μM COR on the maturation of bovine oocytes and development ability of bovine oocytes after vitrification were compared. Finally, the mRNA expression levels of genes associated with lipid synthesis (*SREBP1*, *FABP3*, and *PPARG*) of bovine oocytes, ROS level, mRNA expression levels of genes involved in ER stress (*GRP78*, *XBP1*, and *ATF4*) and mitochondrial function (*MFN1*, *MFN2*, *DRP1*, and *FIS1*), and apoptosis level of vitrified bovine oocytes treated with 1 μM NMN, 2.5 μM BER, or 1 μM COR were analyzed to investigate the relative mechanism.

### 2.9. Statistics Analysis

All experiments were repeated at least three times, and results were presented as mean ± standard deviation. Meanwhile, percentages were subjected to arcsine transformation before statistical analysis. Data were analyzed by one-way analysis of variance (ANOVA) by Duncan’s test using SAS software (SAS Institute, Cary, USA), and *p* < 0.05 was considered statistically significant.

## 3. Result

### 3.1. Effect of NMN, BER, or COR Supplementation during IVM on Lipid Droplet Content in Bovine Oocytes

**Figure 1:**
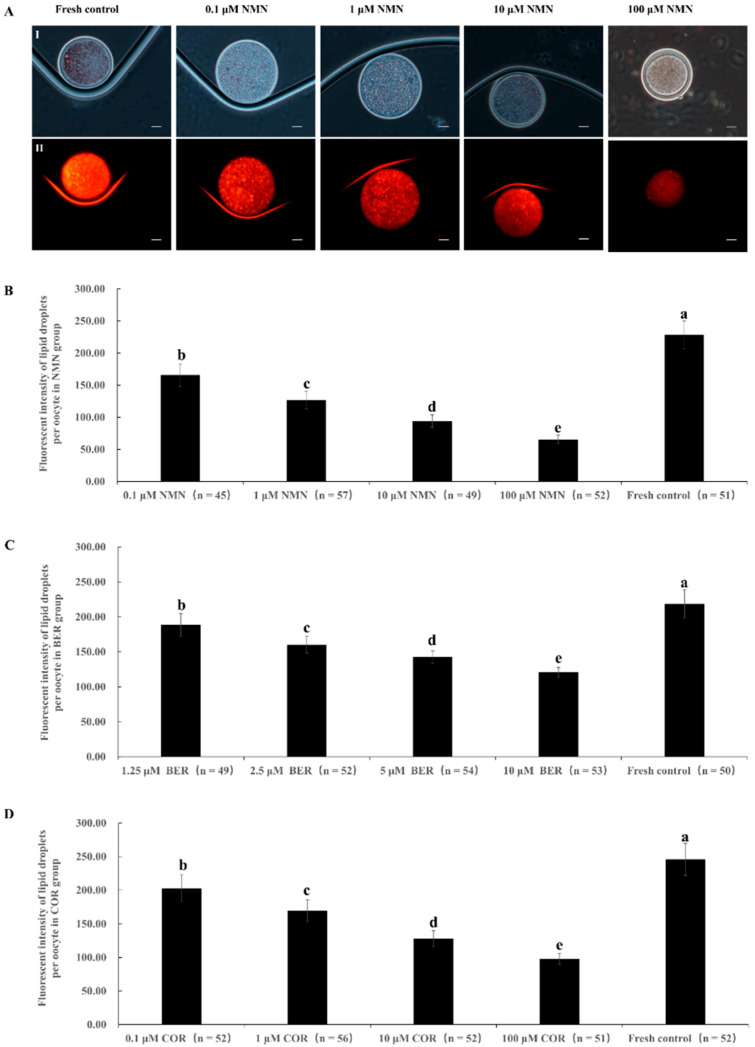
Effect of NMN, BER, or COR on lipid droplet content in fresh bovine oocytes. (**A**) representative images of lipid droplet staining of oocytes. (I) The white light of the oocyte. (II) The image of Nile red staining of lipid droplets. Scale bar = 20 μm. (**B**) effect of different concentrations of NMN on lipid droplet content of bovine oocytes. (**C**) effect of different concentrations of BER on lipid droplet content of bovine oocytes. (**D**): effect of different concentrations of COR on lipid droplet content in bovine oocytes. ^a, b, c, d, e^ Values with different superscripts represent significant differences between groups (*p* < 0.05).

Figure 1A shows representative images of oocyte lipid droplets staining. The results in Figure 1B–D demonstrated that the fluorescence intensity of the lipid droplets in the NMN, BER, and COR groups was obviously decreased compared to the fresh control group (*p* < 0.05). Additionally, the fluorescence intensity significantly decreased as the concentration of NMN, BER, or COR increased.

### 3.2. Effect of NMN Supplementation during IVM on the Maturation Rate of Bovine Oocytes and the Survival Rate and Developmental Ability of Bovine Vitrified Oocytes

Table 2 shows that the percentage of MII oocytes of the 1 μM NMN group (92.24 ± 9.59%) was significantly higher than that of the fresh group (75.42 ± 7.48%, *p* < 0.05). The survival rate of the 1 μM NMN group (97.25 ± 0.11%) was significantly higher than that of vitrification control group (82.29 ± 1.16%, *p* < 0.05). Meanwhile, the cleavage rate and blastocyst rate of the vitrified oocytes in the 1 μM NMN group (75.47 ± 1.11%, 33.75 ± 1.43%) were also higher than those of the vitrification control group (52.00 ± 1.26%, 15.38 ± 1.73%, *p* < 0.05) but still lower than those of the fresh control group (85.60 ± 7.59%, 45.79 ± 3.85%, *p* < 0.05)**.**

### 3.3. Effect of BER Supplementation during IVM on the Maturation Rate of Bovine Oocytes and the Survival Rate and Developmental Ability of Bovine Vitrified Oocytes

Table 3 shows that the percentage of MII oocytes of the 2.5 μM BER group (83.16 ± 8.67%) was significantly higher than that of the fresh group (76.72 ± 7.58%, *p* < 0.05). The survival rate of oocytes after vitrification in the 1.25 μM BER group (89.47 ± 1.64%), 2.5 μM BER group (93.50 ± 0.97%), or 5 μM BER group (89.29 ± 1.39%) was similar to that of the vitrification control group (89.74 ± 1.18%, *p* > 0.05). Meanwhile, the cleavage and blastocyst rates of vitrified oocytes in the 1.25 μM BER group (59.80 ± 0.66%, 16.39 ± 0.92%), 2.5 μM BER group (69.57 ± 1.69%, 25.00 ± 1.80%), or 5 μM BER group (62.00 ± 1.73%, 16.13 ± 4.81%) were also higher than those of the 10 μM BER group (51.76 ± 5.49%, 11.36 ± 4.50%, *p* < 0.05) and the vitrification control group (53.33 ± 1.71%, 12.50 ± 1.26%, *p* < 0.05) but still lower than those of the fresh control group (87.10 ± 7.59%, 46.30 ± 4.13%, *p* < 0.05).

### 3.4. Effect of COR Supplementation during IVM on the Maturation Rate of Bovine Oocytes and the Survival Rate and Developmental Ability of Bovine Vitrified Oocytes

Table 4 shows that the percentage of MII oocytes of the 1 μM COR group (83.63 ± 7.65%) was significantly higher than that of the fresh group (76.07 ± 7.82%, *p* < 0.05). The survival rate of oocytes after vitrification in the 0.1 μM COR group (90.91 ± 1.27%), 1 μM COR group (94.83 ± 0.27%), or 10 μM COR (87.96 ± 2.04%) was similar to that of the vitrification control group (89.19 ± 1.66%, *p* > 0.05). Meanwhile, the cleavage rate and blastocyst rates of vitrified oocytes of the 1 μM COR group (69.09 ± 0.46%, 23.68 ± 1.41%) were also higher than those of the vitrification control group (53.54 ± 2.94%, 13.21 ± 0.59%, *p* < 0.05) but still lower than those of the fresh control group (84.76 ± 6.87%, 43.82 ± 3.59%, *p* < 0.05).

### 3.5. Effect of NMN, BER, or COR Supplementation during IVM on the Maturation Rate of Bovine Oocytes and the Survival Rate and Developmental Ability of Bovine Vitrified Oocytes

Table 5 shows that the percentage of MII oocytes of the 1 μM NMN group (93.08 ± 8.78%), 2.5 μM BER (85.97 ± 9.58%), and 1 μM COR group (83.00 ± 8.46%) was significantly higher than that of the fresh group (75.46 ± 7.68%, *p* < 0.05), the 1 μM NMN group being the highest. The survival rate of oocytes after vitrification in the 1 μM NMN group (98.55 ± 2.25%) was prominently higher than that of the vitrification control group (87.88 ± 7.13%, *p* < 0.05) and 1 μM COR group (86.21 ± 7.06%, *p* < 0.05). Moreover, the cleavage rate and blastocyst rate of vitrified oocytes of the 1 μM NMN group (79.41 ± 5.13%, 31.48 ± 4.79%) were higher than those of the 2.5 μM BER group (68.97 ± 7.34%, 22.50 ± 3.21%, *p* < 0.05) or vitrification control group (50.00 ± 4.47%, 10.34 ± 2.75%, *p* < 0.05). Moreover, the blastocyst rate of vitrified oocytes of the 1 μM NMN group (31.48 ± 4.79%) was higher than the 1 μM COR group (21.05 ± 4.85%, *p* < 0.05) but still lower than that of the fresh control group (42.19 ± 4.10%, *p* < 0.05).

### 3.6. Effect of NMN, BER, or COR Supplementation during IVM on the mRNA Expression Levels of Genes Associated with Lipid Synthesis in Bovine Oocytes

**Figure 2:**
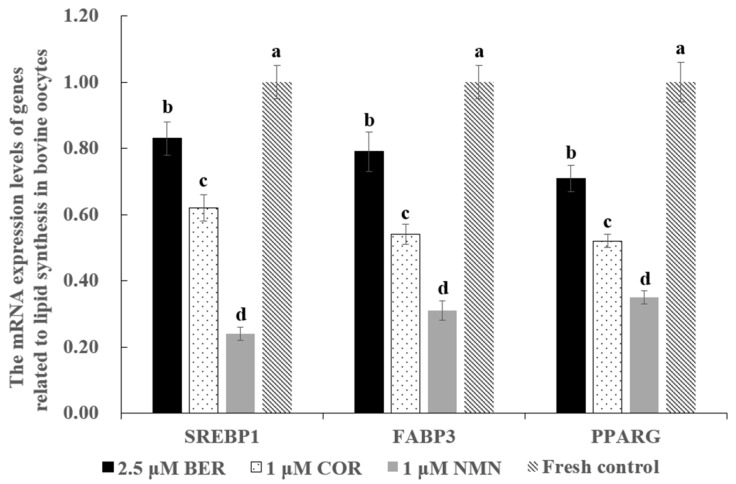
Effect of NMN, BER, or COR supplementation during IVM on the mRNA expression levels of genes related to lipid synthesis in fresh bovine oocytes. ^a, b, c, d^ Values with different superscripts indicate significant differences between groups (*p* < 0.05).

As shown in Figure 2, the mRNA expression levels of genes promoting lipid synthesis (*SREBP1*, *FABP3*, *PPARG*) in the 1 μM NMN, 2.5 μM BER, and 1 μM COR groups were prominently lower than those of the fresh control group (*p* < 0.05), with the 1 μM NMN group being the lowest of all groups.

### 3.7. Effects of NMN, BER, or COR on ROS Level of Bovine Vitrified Oocytes

**Figure 3:**
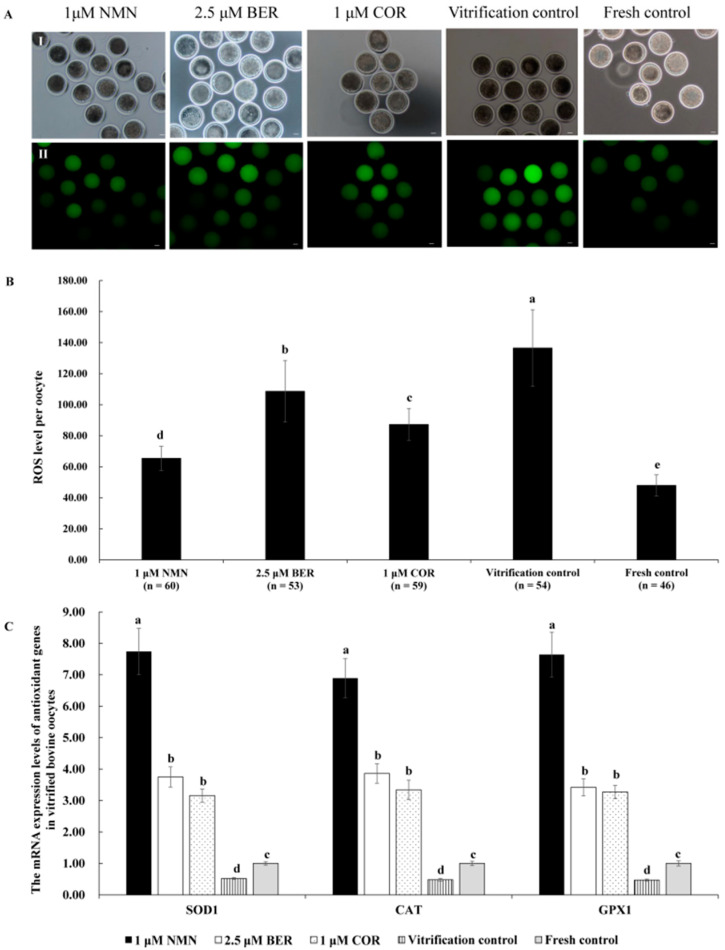
Effect of NMN, BER, and COR on ROS levels in bovine vitrified oocytes. (**A**) representative images of oocyte ROS staining. (I) the white light of the oocyte. (II) the image of DCHFDA staining of oocytes. Scale bar = 20 μm. (**B**) the ROS level of vitrified bovine oocytes. (**C**) the mRNA expression levels of antioxidant genes in bovine vitrified oocytes. ^a, b, c, d, e^ Values with different superscripts represent significant differences between groups (*p* < 0.05).

The representative images of ROS staining are shown in Figure 3A. Figure 3B indicates that the ROS level in vitrified bovine oocytes in the 1 μM NMN group, 2.5 μM BER group, or 1 μM COR group was dramatically lower than that of the vitrification control group (*p* < 0.05) but higher than that of fresh control group (*p* < 0.05), with the 1 μM NMN group being the lowest out of the 1 μM NMN group, 2.5 μM BER group, and 1 μM COR group. Moreover, the results in Figure 3C show that the mRNA expression levels of antioxidant-related genes (*SOD1*, *GPX1*, and *CAT*) in the 1 μM NMN group were significantly higher than those of the 2.5 μM BER group, 1 μM COR group, vitrification control group, and fresh control group (*p* < 0.05).

### 3.8. Effect of NMN, BER, or COR on ER Stress in Bovine Vitrified Oocytes

**Figure 4:**
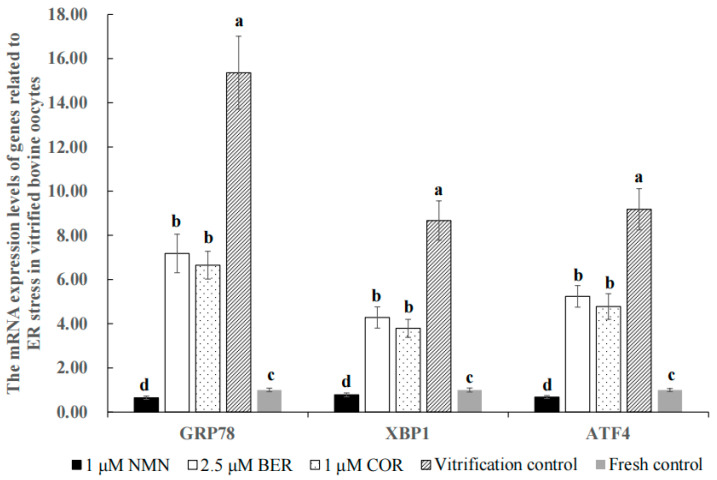
Effect of NMN, BER, or COR on the mRNA expression levels of genes associated with ER stress in bovine vitrified oocytes. ^a, b, c, d^ Values with different superscripts represent significant differences between groups (*p* < 0.05).

Figure 4 shows that the mRNA expression levels of ER stress marker genes (*GRP78*, *XBP1* and *ATF4*) in the 1 μM NMN group, 2.5 μM BER group, 1 μM COR group, and fresh control group were distinctly lower than those of the vitrification control group (*p* < 0.05), with 1 μM NMN being the lowest of all groups (*p* < 0.05).

### 3.9. Effect of NMN, BER, or COR on Mitochondrial Function in Bovine Vitrified Oocytes

**Figure 5:**
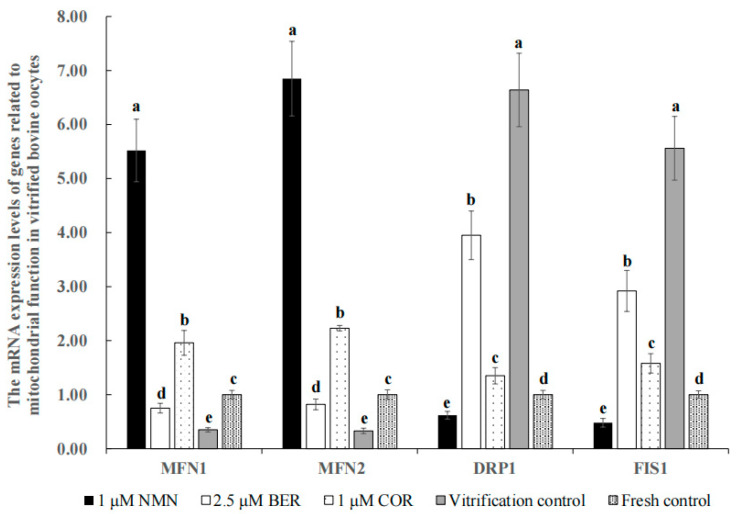
Effect of NMN, BER, or COR on the mRNA expression levels of genes related to mitochondrial function in bovine vitrified oocytes ^a, b, c, d, e^ Values with different superscripts represent significant differences between groups (*p* < 0.05).

Figure 5 shows that the mRNA expression levels of genes (*MFN1* and *MFN2*) promoting mitochondrial fusion in the 1 μM NMN group, 2.5 μM BER group, and 1 μM COR group were distinctly higher than those of the vitrification control group (*p* < 0.05), with the 1 μM NMN group being the highest of all groups. Meanwhile, the mRNA expression levels of genes (*DRP1* and *FIS1*) promoting mitochondrial fission in the 1 μM NMN group, 2.5 μM BER group, and 1 μM COR group were distinctly lower than those of the vitrification control group (*p* < 0.05), with the 1 μM NMN group being the lowest in all groups.

### 3.10. Effects of NMN, BER, or COR on Apoptosis Level of Bovine Vitrified Oocytes

**Figure 6:**
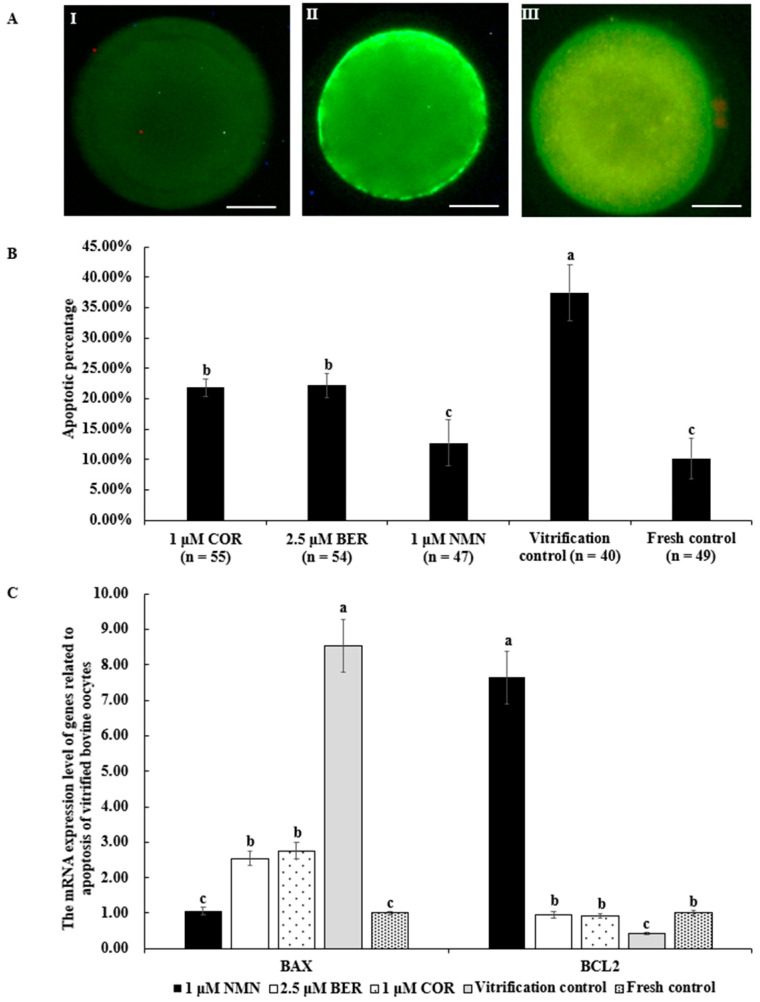
Effect of NMN, BER, or COR on early apoptosis of vitrified bovine oocytes. (**A**) representative images of Annexin V-FITC staining. Scale bar = 20 μm. (I) the images of oocytes with Annexin V-negative signal. (II) the images of oocytes with Annexin V-positive signal. (III) The images of necrotic oocyte. (**B**) the apoptotic percentage of vitrified bovine oocytes. (**C**) the mRNA expression levels of genes related to apoptosis of bovine vitrified oocytes. ^a, b, c^ Values with different superscripts represent significant differences between groups (*p* < 0.05).

The representative images of Annexin V-FITC staining are shown in Figure 6A. The apoptosis percentage of vitrified bovine oocytes in the 1 μM NMN group (12.77 ± 3.73%) was significantly lower than that of the 2.5 μM BER group (22.22 ± 1.99%, *p* < 0.05), 1 μM COR group (21.82 ± 1.38%, *p* < 0.05), and vitrification control group (37.50 ± 4.63%, *p* < 0.05) and comparable to the fresh control group (10.20 ± 3.36%, *p* > 0.05) (Figure 6B). Moreover, the anti-apoptotic gene mRNA expression level of *BCL2* in the 1 μM NMN group was distinctly higher than those of other groups (*p* > 0.05), in contrast to the pro-apoptosis gene expression of *BAX* (Figure 6C).

## 4. Discussion

Wang and colleagues [40] found that after treatment with NMN, the lipid droplet content in mouse oocytes significantly decreased. Similarly, our results in Figure 1B indicated that NMN treatment also led to a significant reduction in lipid droplet content in bovine oocytes, which could be attributed to the reduced expression of genes such as *FABP3*, *SREBP1*, and *PPARG* (as shown in Figure 2). Previous studies have established a positive correlation between the expression levels of *FABP3* [11], *SREBP1* [41], and *PPARG* [42] and lipid accumulation. Wang [43] also reported that NMN could inhibit lipid accumulation by reducing the expression levels of *SREBP1* and *FASN*. According to Uddin [26], NMN also was found to regulate the expression levels of *ACC1*, *MPC1*, and *CD36*, leading to increased lipid absorption and transport. All these findings suggested that NMN could decrease the lipid droplet content of bovine oocytes by reducing the expression levels of *FABP3*, *SREBP1*, and *PPARG*.

As shown in Figure 1C, the lipid droplet content in bovine oocytes was notably reduced in the BER group due to BER’s role in regulating lipid metabolism. BER has previously been demonstrated to decrease lipid accumulation in porcine oocytes [31], colon cancer cells [27], and mouse liver tissue [44] by suppressing the expression of genes that drive lipid buildup, such as *SCAP* [27], *SREBP-1* [27,31], *PPARG* [31], *SCD1*, and *FASN* [45]. Our results, consistent with previous findings, demonstrated that the expression of *FABP3*, *SREBP-1*, and *PPARG* were reduced in bovine oocytes exposed to BER (Figure 2). This reduction in gene expression contributed to the decrease in lipid droplet content observed in oocytes treated with BER.

Our results showed that the lipid droplet content of bovine oocytes was observably reduced in the COR group (Figure 1D). Previous studies have shown that COR has a significant impact on reducing lipid accumulation, which is in line with our findings [24,46,47]. This decrease in lipid accumulation may be attributed to the inhibitory effect of COR on gene expression levels that drive lipid accumulation (Figure 2). In agreement with these findings, Gong [48] found that the expression levels of genes involved in lipid synthesis (*SREBP1-c*, *ACC*, *SCD-1*, and *CD36*), absorption, and transport were significantly reduced by COR. Furthermore, according to Xu [29], the expression of genes responsible for lipid droplet formation such as *FSP27*, *RAB5*, and *RAB11* were also suppressed by COR. These studies collectively help to shed light on the observed decrease in lipid droplet content in oocytes treated with COR.

As shown in Table 2, our findings revealed that 1 μM NMN increased the maturation rate of bovine oocytes. Analogously, NMN could improve the percentage of the polar body extrusion of heat-stressed porcine oocytes via keeping the spindle/chromosome structure, restoring the distribution of cortical granules, and protecting the dynamic polymerization of the actin cytoskeleton [30]. The survival rate of vitrified bovine oocytes improved significantly when treated with 1 μM NMN, which may be attributed to the reduction of lipid droplet content. This observation is supported by previous studies in both bovines [49] and pigs [14], which showed that reducing lipid droplet content can enhance the survival rate of vitrified oocytes. Additionally, our results indicated that the developmental ability of vitrified bovine oocytes treated with 1 μM NMN improved significantly, likely because of the inhibition of ER stress and improvement of mitochondrial function. As shown in Figure 4, our results indicate that the process of vitrification led to a significant increase in the mRNA expression levels of *GRP78*, *XBP1*, and *ATF4*, which are markers of ER stress activation in bovines [50], pigs [51], and mice [52]. Our results also showed that treatment with NMN was able to decrease the expression levels of *GRP78*, *XBP1*, and *ATF4* (Figure 4). This is in line with previous research, which demonstrated that NMN could alleviate heat stress in bovine mammary epithelial cells by reducing ER stress and minimizing mitochondrial damage [19]. Moreover, the balance of mitochondrial fusion and fission is crucial for maintaining mitochondrial quality [19] and an imbalance in this balance can result in a decline in oocyte quality [53,54]. The positive regulation of mitochondrial fusion in mammals is achieved through the genes *MFN1* and *MFN2* [55], while mitochondrial fission is linked to the genes *DRP1* and *FIS1* [56], and inhibition of these genes can inhibit mitochondrial fission [57]. As shown in Figure 5, vitrification caused a disruption in the balance between mitochondrial fusion and fission by inhibiting the expression of *MFN1* and *MFN2* while increasing the expression of *DRP1* and *FIS1*. Similarly, the expression levels of *MFN1*, *MFN2*, *DRP1*, and *FIS1* were also affected by heat stress [58], ischemia [59], vitrification temperatures, and cryoprotective agent concentrations [60]. The results showed that NMN could restore this balance of mitochondrial fusion and fission by upregulating *MFN1* and *MFN2* and downregulating *DRP1* and *FIS1* (Figure 5), similar to previous researches [19,59]. Furthermore, NMN has been proven to reduce the damages of stress such as high-fat diet [40], senescence [33], toxicants [61], and heat stress [30], and improve the quality of oocytes in mice [61] and pigs [30] by reducing meiotic defects, rescuing mitochondrial function, and eliminating the accumulated ROS to suppress apoptosis.

The results in Table 3 indicated that the maturation rate of bovine oocytes was improved by 2.5 μM BER. Analogously, Dai and colleagues [31] also reported this phenomenon in pigs and they also suggested that this was related to BER promoting oocyte lipid metabolism. The use of 2.5 μM BER led to an improvement in both cleavage rate and blastocyst rate of vitrified bovine oocytes, likely due to an enhancement in lipid metabolism. This is in line with previous research which showed that BER improves the maturation rate of porcine oocytes in vitro by activating the expression of miR-192 and promoting lipid metabolism, resulting in an improved cleavage rate and blastocyst rate [31]. Additionally, another study found that the supplementation of 2.5 μM BER significantly promoted the development of two-cell embryos to eight-cell embryos in mice by reducing ROS and apoptosis [34]. Meanwhile, our results showed that the treatment with BER resulted in a reduction in the expression levels of *GRP78*, *XBP1*, *ATF4*, *DRP1*, and *FIS1* and an increase in the expression levels of *MFN1* and *MFN2* (Figure 4 and Figure 5), which suggested that BER reduced the injuries and improved the quality of vitrified oocytes. Similarly, previous research reported that BER could decrease ER stress by decreasing the expression of *ATF4* and *XBP1* [62] and maintain proper mitochondrial morphology by inhibiting the activation of *DRP1* [63] in mammal cells.

As shown in Table 4, our results demonstrated that the maturation rate of bovine oocytes was effectively improved by 1 μM COR. Liu [32] also reported that COR was effective in improving the maturation rate of small porcine oocytes (<3 mm) in vitro [32]. The cleavage rate and blastocyst rate of bovine vitrified oocytes treated with 1 μM COR were effectively improved, which may be due to the reduction of ER stress (Figure 4) and the rescuing of mitochondrial function (Figure 5). Our results align with previous findings that COR has the ability to inhibit the expression of ER stress activation marker genes such as *ATF4* and *CHOP* [64,65], reverse *DRP1*-mediated aberrant mitochondrial fragmentation, and maintain normal mitochondrial morphology [66]. However, other studies have found that the development ability of oocytes in bovines [67,68] and pigs [69] was inhibited by COR in a dose-dependent manner, likely due to the inhibition of polyadenylation at high concentrations of COR. The different results could be explained by the differences in COR concentrations and culture systems used [69].

As shown in Table 5, our results suggested that the blastocyst rate of the 1 μM NMN group was distinctly higher than that of the 2.5 μM BER group and 1 μM COR group, which may be due to the stronger ability of NMN to regulate the gene expression levels of lipid synthesis (Figure 2), antioxidants (Figure 3C), ER stress (Figure 4), mitochondrial function (Figure 5), and anti-apoptosis (Figure 6C).

Previous research has established that vitrification causes a significant increase in the level of ROS in oocytes [70,71], leading to lipid peroxidation and damaging DNA, proteins, and enzyme activity [72]. Similarly, vitrification obviously facilitated the ROS generation of bovine oocytes in our results (Figure 3B). Our results, as depicted in Figure 3B, also demonstrated that NMN, BER, and COR were effective in reducing the level of ROS in vitrified bovine oocytes, as a result of their ability to increase the mRNA expression of antioxidant-related genes (*SOD1*, *GPX1*, and *CAT*) (Figure 3C). Moreover, NMN has been shown to reduce the level of ROS by increasing the activity of *SOD*, which detoxifies ROS, and decreasing the expression levels of *NOX1* and *NOX4* that generate ROS [59,73]. Furthermore, BER reduces the excessive production of mitochondrial ROS by activating the PGC-1α signaling pathway, which supports mitochondrial energy balance [74]. Additionally, COR has also been demonstrated to reduce ROS levels [66,75], due to the increased activity of *SOD* and *CAT* [76]. Furthermore, the ROS level of the NMN group was dramatically lower compared to both the BER group and COR group, which was due to the better capability of NMN to regulate the mRNA expression levels of antioxidant genes (Figure 3C).

Vitrification exposes oocytes to various forms of stress, such as oxidative stress, ER stress, and osmotic stress, ultimately leading to apoptosis [77]. PS externalization, a commonly used marker for apoptosis [78], was also detected in this study. Figure 6 illustrates that vitrification markedly increased the level of apoptosis in vitrified bovine oocytes; however, the addition of NMN, BER, or COR significantly reduced this level, similar to previous research [19,79,80,81,82]. NMN can inhibit apoptosis by reducing the *BAX*/*BCL2* ratio and blocking the cleavage of CASPASE-3 [19]. Similarly, BER reduces apoptosis by suppressing the expression of *BAX*, which is a pro-apoptotic marker gene, and promoting the expression of *BCL2*, which is a marker gene of anti-apoptosis [83]. Additionally, COR reduces apoptosis through inhibiting the pro-apoptotic IRE1-JNK pathway [65]. Furthermore, the level of apoptosis in the 1 μM NMN group was comparable to that of the fresh control group and obviously lower than both the BER group and the COR group, which may be due to NMN’s stronger regulatory effect on genes related to apoptosis (Figure 6C).

## 5. Conclusions

In conclusion, the supplementation of 1 μM NMN, 2.5 μM BER, or 1 μM COR during IVM was found to be effective in reducing the lipid droplet content of bovine oocytes by regulating the expression of lipid metabolism genes and the supplementation also improved the development ability of vitrified bovine oocytes by reducing the generation of ROS, reducing ER stress, regulating mitochondrial function, and inhibiting apoptosis. Furthermore, 1 μM NMN was more effective than 2.5 μM BER and 1 μM COR.

## Figures and Tables

**Table 1:** Primers used in the present study.

Gene	NCBI Accession	Primer (3′-5′)	Size (bp)
*SREBP1*	NM_001113302.1	CTACATCCGCTTCCTTCAGC	90
		TTCAGCGATTTGCTTTTGTG	
*PPARG*	NM_181024.2	ATTTGGAAACGGACGTCTTG	220
		TGAGGTCCTTGCAGACACTG	
*FABP3*	NM_174313.2	TTCAAGCTGGGAGTCGAGTT	235
		GCAGTCAGTGGAAGGAGAGG	
*XBP1*	NM_001034727.3	GACATCCTGTTGGGCATTCT	257
		ACATTGGCTTCGCTCTCTGT	
*ATF4*	NM_001034342.2	AGATGACCTGGAAACCATGC	190
		AGGGGGAAGAGGTTGAAAGA	
*GRP78*	NM_001075148.1	TGGCATTCTTCGAGTGACAG	84
		GTCAGGCGATTTTGGTCATT	
*MFN1*	NM_001206508.1	GCAGACAGCACATGGAAAGA	181
		CTTGCCTGAAATCCTTCTGC	
*MFN2*	NM_001190269.1	GAAGTCGAGAGGCAGGTGTC	131
		CGGTGCAGCTCATTCTTGTA	
*FIS1*	NM_001034784.2	AACGACGACATCCGTAAAGG	238
		CCATGCCCACTAGTCCATCT	
*DRP1*	NM_001046494.2	TGGGGTCTTGTGTGTTACGA	210
		GAGGTCTCCGGGTGACAATA	
*CAT*	NM_001035386.2	TGGGACCCAACTATCTCCAG	178
		AAGTGGGTCCTGTGTTCCAG	
*GPX1*	NM_174076.3	ACATTGAAACCCTGCTGTCC	216
		TCATGAGGAGCTGTGGTCTG	
*SOD1*	NM_174615.2	GGATTCCACGTCCATCAGTT	292
		CAGCGTTGCCAGTCTTTGTA	
*BAX*	NM_173894.1	TCTGACGGCAACTTCAACTG	205
		TGGGTGTCCCAAAGTAGGAG	
*BCL2*	NM_001166486.1	CATCGTGGCCTTCTTTGAGT	111
		CGGTTCAGGTACTCGGTCAT	
*β-ACTIN*	NM_173979.3	ACTTGCGCAGAAAACGAGAT	121
		CACCTTCACCGTTCCAGTTT	

**Table 2:** Effect of NMN supplementation during IVM on the maturation rate of bovine oocytes and the survival rate and developmental ability of bovine vitrified oocytes.

Groups	No. of MII	Survival Rate after Vitrification	Cleavage Rate after IVF	Blastocyst Rate after IVF
0.1 μM NMN	83.38 ± 7.85% (271/325) ^b^	90.57 ± 0.33% (96/106) ^b^	62.50 ± 0.70% (60/96) ^c^	21.67 ± 0.59% (13/60) ^c^
1 μM NMN	92.24 ± 9.59% (321/348) ^a^	97.25 ± 0.11% (106/109) ^a^	75.47 ± 1.11% (80/106) ^b^	33.75 ± 1.43% (27/80) ^b^
10 μM NMN	84.53 ± 8.57% (317/375) ^b^	90.57 ± 1.10% (96/106) ^b^	64.58 ± 0.94% (62/96) ^c^	20.97 ± 1.84% (13/62) ^c^
100 μM NMN	72.01 ± 7.81% (265/368) ^c^	82.00 ± 0.64% (82/100) ^c^	52.44 ± 2.15% (43/82) ^d^	13.95 ± 0.55% (6/43) ^d^
Vitrification control	74.73 ± 7.18% (272/364) ^c^	89.29 ± 1.16% (100/112) ^b^	52.00 ± 1.26% (52/100) ^d^	15.38 ± 1.73% (8/52) ^d^
Fresh control	75.42 ± 7.48% (267/354) ^c^	-	85.60 ± 7.59% (107/125) ^a^	45.79 ± 3.85% (49/107) ^a^

^a, b, c, d^ Values with different superscripts represent significant differences between groups (*p* < 0.05).

**Table 3:** Effect of BER supplementation during IVM on the maturation rate of bovine oocytes and the survival rate and developmental ability of bovine vitrified oocytes.

Groups	No. of MII	Survival Rate after Vitrification	Cleavage Rate after IVF	Blastocyst Rate after IVF
1.25 μM BER	78.64 ± 9.52% (313/398) ^ab^	89.47 ± 1.64% (102/114) ^a^	59.80 ± 0.66% (61/102) ^c^	16.39 ± 0.92% (10/61) ^c^
2.5 μM BER	83.16 ± 8.67% (321/386) ^a^	93.50 ± 0.97% (115/123) ^a^	69.57 ± 1.69% (80/115) ^b^	25.00 ± 1.80% (20/80) ^b^
5 μM BER	79.43 ± 8.12% (305/384) ^ab^	89.29 ± 1.39% (100/112) ^a^	62.00 ± 1.73% (62/100) ^c^	16.13 ± 4.81% (10/62) ^c^
10 μM BER	72.96 ± 6.87% (286/392) ^c^	81.73 ± 3.21% (85/104) ^b^	51.76 ± 5.49% (44/85) ^d^	11.36 ± 4.50% (5/44) ^d^
Vitrification control	75.32 ± 7.62% (293/389) ^bc^	89.74 ± 1.18% (105/117) ^a^	53.33 ± 1.71% (56/105) ^d^	12.50 ± 1.26% (7/56) ^d^
Fresh control	76.72 ± 7.58% (290/378) ^bc^	-	87.10 ± 7.59% (108/124) ^a^	46.30 ± 4.13% (50/108) ^a^

^a, b, c, d^ Values with different superscripts represent significant differences between groups (*p* < 0.05).

**Table 4:** Effect of COR supplementation during IVM on the maturation rate of bovine oocytes and the survival rate and developmental ability of bovine vitrified oocytes.

Groups	No. of MII	Survival Rate after Vitrification	Cleavage Rate after IVF	Blastocyst Rate after IVF
0.1 μM COR	79.49 ±8.45 % (248/312) ^ab^	90.91 ± 1.27% (100/110) ^ab^	60.00 ± 3.20% (60/100) ^c^	15.00 ± 0.75% (9/60) ^c^
1 μM COR	83.63 ± 7.65% (286/342) ^a^	94.83 ± 0.27% (110/116) ^a^	69.09 ± 0.46% (76/110) ^b^	23.68 ± 1.41% (18/76) ^b^
10 μM COR	79.40 ± 7.49% (266/335) ^ab^	87.96 ± 2.04% (95/108) ^b^	60.00 ± 0.00% (57/95) ^c^	15.79 ± 1.37% (9/57) ^c^
100 μM COR	71.64 ± 6.98% (245/342) ^c^	71.00 ± 9.94% (71/100) ^c^	47.89 ± 4.38% (34/71) ^e^	11.76 ± 0.80% (4/34) ^d^
Vitrification control	75.79 ± 7.49% (241/318) ^bc^	89.19 ± 1.66% (99/111) ^ab^	53.54 ± 2.94% (53/99) ^d^	13.21 ± 0.59% (7/53) ^cd^
Fresh control	76.07 ± 7.82% (248/326) ^bc^	-	84.76 ± 6.87% (89/105) ^a^	43.82 ± 3.59% (39/89) ^a^

^a, b, c, d, e^ Values with different superscripts represent significant differences between groups (*p* < 0.05).

**Table 5:** Effect of NMN, BER, or COR supplementation during IVM on the maturation rate of bovine oocytes and the survival rate and developmental ability of bovine vitrified oocytes.

Groups	No. of MII	Survival Rate after vitrification	Cleavage Rate after IVF	Blastocyst Rate after IVF
2.5 μM BER	85.97 ± 9.58 % (239/278) ^b^	93.55 ± 2.71% (58/62) ^ab^	68.97 ± 7.34% (40/58) ^c^	22.50 ± 3.21% (9/40) ^c^
1 μM COR	83.00 ± 8.46% (205/247) ^b^	86.21 ± 7.06% (75/87) ^b^	76.00 ± 7.23% (57/75) ^b^	21.05 ± 4.85% (12/57) ^c^
1 μM NMN	93.08 ± 8.78% (269/289) ^a^	98.55 ± 2.25% (68/69) ^a^	79.41 ± 5.13% (54/68) ^ab^	31.48 ± 4.79% (17/54) ^b^
Vitrification control	73.23 ± 7.29% (186/254) ^c^	87.88 ± 7.13% (58/66) ^b^	50.00 ± 4.47% (29/58) ^d^	10.34 ± 2.75% (3/29) ^d^
Fresh control	75.46 ± 7.68% (203/269) ^c^	-	82.05 ± 5.83% (64/78) ^a^	42.19 ± 4.10% (27/64) ^a^

^a, b, c, d^ Values with different superscripts represent significant differences between groups (*p* < 0.05).

## Data Availability

All the data supporting the conclusions in this article have been presented in the manuscript.

## References

[1] Sprícigo J.F., Morais K.S., Yang B.S., Dode M.A. (2012). Effect of the exposure to methyl-β-cyclodextrin prior to chilling or vitrification on the viability of bovine immature oocytes. Cryobiology.

[2] Morrell J.M., Mayer I. (2017). Reproduction biotechnologies in germplasm banking of livestock species: A review. Zygote.

[3] Mayer I. (2019). The Role of Reproductive sciences in the preservation and breeding of commercial and threatened teleost fishes. Adv. Exp. Med. Biol..

[4] Vining L.M., Zak L.J., Harvey S.C., Harvey K.E. (2021). The role of apoptosis in cryopreserved animal oocytes and embryos. Theriogenology.

[5] Telfer E.E., Andersen C.Y. (2021). In vitro growth and maturation of primordial follicles and immature oocytes. Fertil. Steril..

[6] Mullen S.F., Fahy G.M. (2012). A chronologic review of mature oocyte vitrification research in cattle, pigs, and sheep. Theriogenology.

[7] Ortiz-Escribano N., Bogado Pascottini O., Woelders H., Vandenberghe L., De Schauwer C., Govaere J., Van den Abbeel E., Vullers T., Ververs C., Roels K. (2018). An improved vitrification protocol for equine immature oocytes, resulting in a first live foal. Equine Vet. J..

[8] Yodrug T., Parnpai R., Hirao Y., Somfai T. (2021). Effect of vitrification at different meiotic stages on epigenetic characteristics of bovine oocytes and subsequently developing embryos. Anim. Sci. J..

[9] McEvoy T.G., Coull G.D., Broadbent P.J., Hutchinson J.S., Speake B.K. (2000). Fatty acid composition of lipids in immature cattle, pig and sheep oocytes with intact zona pellucida. J. Reprod. Fertil..

[10] Yang X., Wu L.L., Chura L.R., Liang X., Lane M., Norman R.J., Robker R.L. (2012). Exposure to lipid-rich follicular fluid is associated with endoplasmic reticulum stress and impaired oocyte maturation in cumulus-oocyte complexes. Fertil. Steril..

[11] Del Collado M., da Silveira J.C., Sangalli J.R., Andrade G.M., Sousa L., Silva L.A., Meirelles F.V., Perecin F. (2017). Fatty acid binding protein 3 and transzonal projections are involved in lipid accumulation during in vitro maturation of bovine oocytes. Sci. Rep..

[12] Quan G., Wu G., Hong Q. (2017). Oocyte Oryopreservation based in sheep: The current status and future perspective. Biopreserv. Biobank..

[13] Amstislavsky S., Mokrousova V., Brusentsev E., Okotrub K., Comizzoli P. (2019). Influence of cellular lipids on cryopreservation of mammalian oocytes and preimplantation embryos: A review. Biopreserv. Biobank..

[14] Hara K., Abe Y., Kumada N., Aono N., Kobayashi J., Matsumoto H., Sasada H., Sato E. (2005). Extrusion and removal of lipid from the cytoplasm of porcine oocytes at the germinal vesicle stage: Centrifugation under hypertonic conditions influences vitrification. Cryobiology.

[15] Owen C.M., Johnson M.A., Rhodes-Long K.A., Gumber D.J., Barceló-Fimbres M., Altermatt J.L., Campos-Chillon L.F. (2022). Novel synthetic oviductal fluid for conventional freezing 1 (SCF1) culture medium improves development and cryotolerance of in vitro produced holstein embryos. J. Anim. Sci..

[16] Ren L., Fu B., Ma H., Liu D. (2015). Effects of mechanical delipation in porcine oocytes on mitochondrial distribution, ROS activity and viability after vitrification. CryoLetters.

[17] Poddar S.K., Sifat A.E., Haque S., Nahid N.A., Chowdhury S., Mehedi I. (2019). Nicotinamide mononucleotide: Exploration of diverse therapeutic applications of a potential molecule. Biomolecules.

[18] Zhong O., Wang J., Tan Y., Lei X., Tang Z. (2022). Effects of NAD+ precursor supplementation on glucose and lipid metabolism in humans: A meta-analysis. Nutr. Metab..

[19] Zeng H.F., Xu J., Wang X.L., Li S.J., Han Z.Y. (2022). Nicotinamide mononucleotide alleviates heat stress-induced oxidative stress and apoptosis in BMECs through reducing mitochondrial damage and endoplasmic reticulum stress. Ecotoxicol. Environ. Saf..

[20] Tian Y., Zhu C.L., Li P., Li H.R., Liu Q., Deng X.M., Wang J.F. (2023). Nicotinamide mononucleotide attenuates LPS-Induced acute lung injury with anti-inflammatory, anti-oxidative and anti-apoptotic effects. J. Surg. Res..

[21] Hong W., Mo F., Zhang Z., Huang M., Wei X. (2020). Nicotinamide mononucleotide: A promising molecule for therapy of diverse diseases by targeting NAD+ metabolism. Front. Cell. Dev. Biol..

[22] Okabe K., Yaku K., Tobe K., Nakagawa T. (2019). Implications of altered NAD metabolism in metabolic disorders. J. Biomed. Sci..

[23] Cicero A.F., Baggioni A. (2016). Berberine and its role in chronic disease. Adv. Exp. Med. Biol..

[24] Li T., Wen L., Cheng B. (2019). Cordycepin alleviates hepatic lipid accumulation by inducing protective autophagy via PKA/mTOR pathway. Biochem. Biophys. Res. Commun..

[25] Han F., Dou M., Wang Y., Xu C., Li Y., Ding X., Xue W., Zheng J., Tian P., Ding C. (2020). Cordycepin protects renal ischemia/reperfusion injury through regulating inflammation, apoptosis, and oxidative stress. Acta Biochim. Biophys. Sin..

[26] Uddin G.M., Youngson N.A., Chowdhury S.S., Hagan C., Sinclair D.A., Morris M.J. (2020). Administration of nicotinamide mononucleotide (NMN) reduces metabolic impairment in male mouse offspring from obese mothers. Cells.

[27] Liu Y., Hua W., Li Y., Xian X., Zhao Z., Liu C., Zou J., Li J., Fang X., Zhu Y. (2020). Berberine suppresses colon cancer cell proliferation by inhibiting the SCAP/SREBP-1 signaling pathway-mediated lipogenesis. Biochem. Pharmacol..

[28] Niu Y.J., Tao R.Y., Liu Q., Tian J.Y., Ye F., Zhu P., Zhu H.B. (2010). Improvement on lipid metabolic disorder by 3′-deoxyadenosine in high-fat-diet-induced fatty mice. Am. J. Chin. Med..

[29] Xu H., Wu B., Wang X., Ma F., Li Y., An Y., Wang C., Wang X., Luan W., Li S. (2019). Cordycepin regulates body weight by inhibiting lipid droplet formation, promoting lipolysis and recruiting beige adipocytes. J. Pharm. Pharmacol..

[30] Song M., Li Y., Zhou Y., Yan J., Zhou X., Gao Q., Miao Y., Xiong B. (2022). Nicotinamide mononucleotide supplementation improves the quality of porcine oocytes under heat stress. J. Anim. Sci. Biotechnol..

[31] Dai J., Huang X., Zhang C., Luo X., Cao S., Wang J., Liu B., Gao J. (2021). Berberine regulates lipid metabolism via miR-192 in porcine oocytes matured in vitro. Vet. Med. Sci..

[32] Liu H., Gao Y., Zhai B., Jiang H., Ding Y., Zhang L., Li C., Deng Q., Yu X., Zhang J. (2016). The effects of polyadenylation status on MPFs during in vitro porcine oocyte maturation. Cell Physiol. Biochem..

[33] Miao Y., Cui Z., Gao Q., Rui R., Xiong B. (2020). Nicotinamide mononucleotide supplementation reverses the declining quality of maternally aged oocytes. Cell Rep..

[34] Huang C.H., Wang F.T., Chan W.H. (2020). Dose-dependent beneficial and harmful effects of berberine on mouse oocyte maturation and fertilization and fetal development. Toxicol. Res..

[35] Zhao X.M., Hao H.S., Du W.H., Zhao S.J., Wang H.Y., Wang N., Wang D., Liu Y., Qin T., Zhu H.B. (2016). Melatonin inhibits apoptosis and improves the developmental potential of vitrified bovine oocytes. J. Pineal Res..

[36] Brackett B.G., Oliphant G. (1975). Capacitation of rabbit spermatozoa in vitro. Biol. Reprod..

[37] Schmittgen T.D., Livak K.J. (2008). Analyzing real-time PCR data by the comparative C(T) method. Nat. Protoc..

[38] Buschiazzo J., Ríos G.L., Canizo J.R., Antollini S.S., Alberio R.H. (2017). Free cholesterol and cholesterol esters in bovine oocytes: Implications in survival and membrane raft organization after cryopreservation. PLoS ONE.

[39] Anguita B., Vandaele L., Mateusen B., Maes D., Van Soom A. (2007). Developmental competence of bovine oocytes is not related to apoptosis incidence in oocytes, cumulus cells and blastocysts. Theriogenology.

[40] Wang L., Chen Y., Wei J., Guo F., Li L., Han Z., Wang Z., Zhu H., Zhang X., Li Z. (2022). Administration of nicotinamide mononucleotide improves oocyte quality of obese mice. Cell Prolif..

[41] Li L., Zhang X., Ren H., Huang X., Shen T., Tang W., Dou L., Li J. (2021). miR-23a/b-3p promotes hepatic lipid accumulation by regulating Srebp-1c and Fas. J. Mol. Endocrinol..

[42] Zhuang J.L., Liu Y.Y., Li Z.Z., Zhuang Q.Z., Tang W.Z., Xiong Y., Huang X.Z. (2021). Amentoflavone prevents ox-LDL-induced lipid accumulation by suppressing the PPARγ/CD36 signal pathway. Toxicol. Appl. Pharmacol..

[43] Wang L.F., Wang X.N., Huang C.C., Hu L., Xiao Y.F., Guan X.H., Qian Y.S., Deng K.Y., Xin H.B. (2017). Inhibition of NAMPT aggravates high fat diet-induced hepatic steatosis in mice through regulating Sirt1/AMPKα/SREBP1 signaling pathway. Lipids Health Dis..

[44] Xu X., Zhu X.P., Bai J.Y., Xia P., Li Y., Lu Y., Li X.Y., Gao X. (2019). Berberine alleviates nonalcoholic fatty liver induced by a high-fat diet in mice by activating SIRT3. FASEB J..

[45] Ke X., Zhang R., Li P., Zuo L., Wang M., Yang J., Wang J. (2022). Hydrochloride berberine ameliorates alcohol-induced liver injury by regulating inflammation and lipid metabolism. Biochem. Biophys. Res. Commun..

[46] An Y., Li Y., Wang X., Chen Z., Xu H., Wu L., Li S., Wang C., Luan W., Wang X. (2018). Cordycepin reduces weight through regulating gut microbiota in high-fat diet-induced obese rats. Lipids Health Dis..

[47] Wu C., Guo Y., Su Y., Zhang X., Luan H., Zhang X., Zhu H., He H., Wang X., Sun G. (2014). Cordycepin activates AMP-activated protein kinase (AMPK) via interaction with the γ1 subunit. J. Cell Mol. Med..

[48] Gong X., Li T., Wan R., Sha L. (2021). Cordycepin attenuates high-fat diet-induced non-alcoholic fatty liver disease via down-regulation of lipid metabolism and inflammatory responses. Int. Immunopharmacol..

[49] Chankitisakul V., Somfai T., Inaba Y., Techakumphu M., Nagai T. (2013). Supplementation of maturation medium with L-carnitine improves cryo-tolerance of bovine in vitro matured oocytes. Theriogenology.

[50] Khatun H., Wada Y., Konno T., Tatemoto H., Yamanaka K.I. (2020). Endoplasmic reticulum stress attenuation promotes bovine oocyte maturation in vitro. Reproduction.

[51] Park H.J., Park J.Y., Kim J.W., Yang S.G., Jung J.M., Kim M.J., Kang M.J., Cho Y.H., Wee G., Yang H.Y. (2018). Melatonin improves the meiotic maturation of porcine oocytes by reducing endoplasmic reticulum stress during in vitro maturation. J. Pineal Res..

[52] Wu L.L., Russell D.L., Norman R.J., Robker R.L. (2012). Endoplasmic reticulum (ER) stress in cumulus-oocyte complexes impairs pentraxin-3 secretion, mitochondrial membrane potential (DeltaPsi m), and embryo development. Mol. Endocrinol..

[53] Li C.J., Lin L.T., Tsai H.W., Wen Z.H., Tsui K.H. (2022). Phosphoglycerate mutase family member 5 maintains oocyte quality via mitochondrial dynamic rearrangement during aging. Aging Cell.

[54] Jiang X., Xu X., Wang B., Song K., Zhang J., Chen Y., Tian Y., Weng J., Liang Y., Ma W. (2023). Adverse effects of 2-methoxyestradiol on mouse oocytes during reproductive aging. Chem. Biol. Interact..

[55] Chen H., Chomyn A., Chan D.C. (2005). Disruption of fusion results in mitochondrial heterogeneity and dysfunction. J. Biol. Chem..

[56] Smirnova E., Griparic L., Shurland D.L., van der Bliek A.M. (2001). Dynamin-related protein Drp1 is required for mitochondrial division in mammalian cells. Mol. Biol. Cell..

[57] Kim D., Sankaramoorthy A., Roy S. (2020). Downregulation of Drp1 and Fis1 inhibits mitochondrial fission and prevents high glucose-induced apoptosis in retinal endothelial Cells. Cells.

[58] Chen K.L., Wang H.L., Jiang L.Z., Qian Y., Yang C.X., Chang W.W., Zhong J.F., Xing G.D. (2020). Heat stress induces apoptosis through disruption of dynamic mitochondrial networks in dairy cow mammary epithelial cells. In Vitro Cell. Dev. Biol. Anim..

[59] Klimova N., Fearnow A., Long A., Kristian T. (2020). NAD(+) precursor modulates post-ischemic mitochondrial fragmentation and reactive oxygen species generation via SIRT3 dependent mechanisms. Exp. Neurol..

[60] Zhang Z.Y., Yu X.L., Cai M.D., Liu Y.H., Liu J.Q., Zhao S.Y., Li X.X., Li Y.H. (2020). Relationship between bovine oocytes developmental competence and mRNA expression of apoptotic and mitochondrial genes following the change of vitrification temperatures and cryoprotectant concentrations. Cryobiology.

[61] Miao Y., Li X., Shi X., Gao Q., Chen J., Wang R., Fan Y., Xiong B. (2021). Nicotinamide mononucleotide restores the meiotic competency of porcine oocytes exposed to ethylene glycol butyl ether. Front. Cell Dev. Biol..

[62] Xu M., Qi Q., Men L., Wang S., Li M., Xiao M., Chen X., Wang S., Wang G., Jia H. (2020). Berberine protects Kawasaki disease-induced human coronary artery endothelial cells dysfunction by inhibiting of oxidative and endoplasmic reticulum stress. Vasc. Pharmacol..

[63] Qin X., Zhao Y., Gong J., Huang W., Su H., Yuan F., Fang K., Wang D., Li J., Zou X. (2019). Berberine protects glomerular podocytes via inhibiting Drp1-mediated mitochondrial fission and dysfunction. Theranostics.

[64] Jin M.L., Park S.Y., Kim Y.H., Oh J.I., Lee S.J., Park G. (2014). The neuroprotective effects of cordycepin inhibit glutamate-induced oxidative and ER stress-associated apoptosis in hippocampal HT22 cells. Neurotoxicology.

[65] Kitamura M., Kato H., Saito Y., Nakajima S., Takahashi S., Johno H., Gu L., Katoh R. (2011). Aberrant, differential and bidirectional regulation of the unfolded protein response towards cell survival by 3′-deoxyadenosine. Cell Death Differ..

[66] Zhang X.L., Huang W.M., Tang P.C., Sun Y., Zhang X., Qiu L., Yu B.C., Zhang X.Y., Hong Y.X., He Y. (2021). Anti-inflammatory and neuroprotective effects of natural cordycepin in rotenone-induced PD models through inhibiting Drp1-mediated mitochondrial fission. Neurotoxicology.

[67] Krischek C., Meinecke B. (2002). In vitro maturation of bovine oocytes requires polyadenylation of mRNAs coding proteins for chromatin condensation, spindle assembly, MPF and MAP kinase activation. Anim. Reprod. Sci..

[68] Traverso J.M., Donnay I., Lequarre A.S. (2005). Effects of polyadenylation inhibition on meiosis progression in relation to the polyadenylation status of cyclins A2 and B1 during in vitro maturation of bovine oocytes. Mol. Reprod. Dev..

[69] Zhang D.X., Cui X.S., Kim N.H. (2009). Involvement of polyadenylation status on maternal gene expression during in vitro maturation of porcine oocytes. Mol. Reprod. Dev..

[70] Shirzeyli M.H., Eini F., Shirzeyli F.H., Majd S.A., Ghahremani M., Joupari M.D., Novin M.G. (2021). Assessment of mitochondrial function and developmental potential of mouse oocytes after mitoquinone supplementation during vitrification. J. Am. Assoc. Lab. Anim. Sci..

[71] García-Martínez T., Vendrell-Flotats M., Martínez-Rodero I., Ordóñez-León E.A., Álvarez-Rodríguez M., López-Béjar M., Yeste M., Mogas T. (2020). Glutathione ethyl ester protects in vitro-maturing bovine oocytes against oxidative stress induced by subsequent vitrification/warming. Int. J. Mol. Sci..

[72] Su L.J., Zhang J.H., Gomez H., Murugan R., Hong X., Xu D., Jiang F., Peng Z.Y. (2019). Reactive oxygen species-induced lipid peroxidation in apoptosis, autophagy, and ferroptosis. Oxid. Med. Cell Longev..

[73] Yamaura K., Mifune Y., Inui A., Nishimoto H., Kurosawa T., Mukohara S., Hoshino Y., Niikura T., Kuroda R. (2022). Antioxidant effect of nicotinamide mononucleotide in tendinopathy. BMC Musculoskelet. Disord..

[74] Qin X., Jiang M., Zhao Y., Gong J., Su H., Yuan F., Fang K., Yuan X., Yu X., Dong H. (2020). Berberine protects against diabetic kidney disease via promoting PGC-1α-regulated mitochondrial energy homeostasis. Br. J. Pharmacol..

[75] Ku C.W., Ho T.J., Huang C.Y., Chu P.M., Ou H.C., Hsieh P.L. (2021). Cordycepin attenuates palmitic acid-induced inflammation and apoptosis of vascular endothelial cells through mediating PI3K/Akt/eNOS signaling pathway. Am. J. Chin. Med..

[76] Sun H., Zhang A., Gong Y., Sun W., Yan B., Lei S., Yao L.H. (2022). Improving effect of cordycepin on insulin synthesis and secretion in normal and oxidative-damaged INS-1 cells. Eur. J. Pharmacol..

[77] Chang C.C., Shapiro D.B., Nagy Z.P. (2022). The effects of vitrification on oocyte quality. Biol. Reprod..

[78] Casao A., Mendoza N., Pérez-Pé R., Grasa P., Abecia J.A., Forcada F., Cebrián-Pérez J.A., Muino-Blanco T. (2010). Melatonin prevents capacitation and apoptotic-like changes of ram spermatozoa and increases fertility rate. J. Pineal Res..

[79] Pu Q., Guo X.X., Hu J.J., Li A.L., Li G.G., Li X.Y. (2022). Nicotinamide mononucleotide increases cell viability and restores tight junctions in high-glucose-treated human corneal epithelial cells via the SIRT1/Nrf2/HO-1 pathway. Biomed. Pharmacother..

[80] Wu Y.Z., Zhang L., Wu Z.X., Shan T.T., Xiong C. (2019). Berberine ameliorates doxorubicin-induced cardiotoxicity via a SIRT1/p66Shc-mediated pathway. Oxid. Med. Cell Longev..

[81] Song T., Chen W.D. (2021). Berberine inhibited carotid atherosclerosis through PI3K/AKTmTOR signaling pathway. Bioengineered.

[82] Song H., Huang L.P., Li Y., Liu C., Wang S., Meng W., Wei S., Liu X.P., Gong Y., Yao L.H. (2018). Neuroprotective effects of cordycepin inhibit Aβ-induced apoptosis in hippocampal neurons. Neurotoxicology.

[83] Liu K., Sun T., Luan Y., Chen Y., Song J., Ling L., Yuan P., Li R., Cui K., Ruan Y. (2022). Berberine ameliorates erectile dysfunction in rats with streptozotocin-induced diabetes mellitus through the attenuation of apoptosis by inhibiting the SPHK1/S1P/S1PR2 and MAPK pathways. Andrology.

